# Enzymatic Analysis of Recombinant Japanese Encephalitis Virus NS2B(H)-NS3pro Protease with Fluorogenic Model Peptide Substrates

**DOI:** 10.1371/journal.pone.0036872

**Published:** 2012-05-15

**Authors:** Muhammad Junaid, Chakard Chalayut, Anna Sehgelmeble Torrejon, Chanan Angsuthanasombat, Iryna Shutava, Maris Lapins, Jarl E. S. Wikberg, Gerd Katzenmeier

**Affiliations:** 1 Laboratory of Molecular and Cellular Microbiology, Institute of Molecular Biosciences, Mahidol University, Salaya, Nakornpathom, Thailand; 2 Department of Pharmaceutical Biosciences, Division of Pharmacology, Uppsala University, Uppsala, Sweden; Centro de Biología Molecular Severo Ochoa (CSIC-UAM), Spain

## Abstract

**Background:**

Japanese encephalitis virus (JEV), a member of the *Flaviviridae* family, causes around 68,000 encephalitis cases annually, of which 20–30% are fatal, while 30–50% of the recovered cases develop severe neurological sequelae. Specific antivirals for JEV would be of great importance, particularly in those cases where the infection has become persistent. Being indispensable for flaviviral replication, the NS2B-NS3 protease is a promising target for design of anti-flaviviral inhibitors. Contrary to related flaviviral proteases, the JEV NS2B-NS3 protease is structurally and mechanistically much less characterized. Here we aimed at establishing a straightforward procedure for cloning, expression, purification and biochemical characterization of JEV NS2B(H)-NS3pro protease.

**Methodology/Principal Findings:**

The full-length sequence of JEV NS2B-NS3 genotype III strain JaOArS 982 was obtained as a synthetic gene. The sequence of NS2B(H)-NS3pro was generated by splicing by overlap extension PCR (SOE-PCR) and cloned into the pTrcHisA vector. Hexahistidine-tagged NS2B(H)-NS3pro, expressed in *E. coli* as soluble protein, was purified to >95% purity by a single-step immobilized metal affinity chromatography. SDS-PAGE and immunoblotting of the purified enzyme demonstrated NS2B(H)-NS3pro precursor and its autocleavage products, NS3pro and NS2B(H), as 36, 21, and 10 kDa bands, respectively. Kinetic parameters, *K*_m_ and *k*_cat_, for fluorogenic protease model substrates, Boc-GRR-amc, Boc-LRR-amc, Ac-nKRR-amc, Bz-nKRR-amc, Pyr-RTKR-amc and Abz-(R)_4_SAG-nY-amide, were obtained using inner filter effect correction. The highest catalytic efficiency *k*_cat_ */K*_m_ was found for Pyr-RTKR-amc (*k*_cat_/*K*_m_: 1962.96±85.0 M^−1^ s^−1^) and the lowest for Boc-LRR-amc (*k*_cat_/*K*_m_: 3.74±0.3 M^−1^ s^−1^). JEV NS3pro is inhibited by aprotinin but to a lesser extent than DEN and WNV NS3pro.

**Conclusions/Significance:**

A simplified procedure for the cloning, overexpression and purification of the NS2B(H)-NS3pro was established which is generally applicable to other flaviviral proteases. Kinetic parameters obtained for a number of model substrates and inhibitors, are useful for the characterization of substrate specificity and eventually for the design of high-throughput assays aimed at antiviral inhibitor discovery.

## Introduction

Japanese encephalitis virus (JEV) is a mosquito borne flavivirus that causes severe central nervous system diseases such as an acute flaccid paralysis, aseptic meningitis and encephalitis [1]. It belongs to the genus Flavivirus (family *Flaviviridae)* which comprises over 70 viruses many of which are human pathogens, including West Nile virus (WNV), Dengue virus (DEN), Yellow fever virus (YFV), Murray Valley Encephalitis Virus (MVEV), Kunjin Virus (KUNV) and Tick-Borne Encephalitis Virus (TBEV) [2]. Japanese encephalitis (JE) is among the most important viral encephalitides in Asia [3]–[5]. Moreover, the disease is not restricted to Asia and cases also occur sporadically in northern Australia and western Pacific [6]. Of about 68,000 estimated annual cases, approximately 20–30% are fatal, and 30–50% of survivors have significant neurologic sequelae [7], [8]. Since the zoonosis is endemic in large parts of Asia, it is not likely to ever be extinguished.

Currently there is no antiviral therapy for JEV or any other flaviviral infection, and so far the main strategy to control the incidence is by preventive methods such as vaccination and preventing mosquito bites [9]–[11]. Although the improvements in JEV vaccination coverage has reduced the JE incidence, about 55,000 (81%) out of the total annual cases still occur in areas with well established or developing JE vaccination programs [12].

Effective antiviral therapy is thus urgently needed, especially for those cases where the infection has become persistent. One approach to develop anti-JEV therapy is to interfere with the life cycle of the virus, and exploit the molecular targets such as envelope glycoprotein, NS3 protease, NS3 helicase, NS5 methyltransferase and NS5 RNA- dependent RNA polymerase [13]. Unlike several other flaviviruses such as DEN, WNV and MVEV [14]–[16] whose protease enzymes are extensively characterised as potential drug targets, the JEV protease is comparatively less studied with a view to structure-activity relations.

The JEV two-component protease NS2B/NS3 is responsible for processing the viral polyprotein precursor to the mature viral proteins involved in viral pathogenesis, and therefore considered an important drug target in JEV [17], [18]. The N-terminal one-third (180 residues) of NS3 represents the protease domain NS3(pro) that works in coordination with the C-terminal two-third portion RNA helicase during viral propagation [19], [20]. The proteolytic domain contains a classical catalytic triad of H51, D75 and S135, and autocatalytic proteolytic cleavage at the NS2B/NS3 polyprotein junction leads to the formation of a non-covalent complex of NS2B and NS3 [21]. Earlier studies have revealed that a 35–48 amino acid residues long central hydrophilic region NS2B(H) of NS2B interacts directly with the NS3(pro) and promotes folding of NS3(pro) into a catalytically competent conformation [22]–[25].

Currently, there is no X-ray crystallographic structure available for the JEV protease, but crystal structures of the similar proteases from DEN and WNV have provided insight into the mechanism of cofactor-dependent activation and revealed an ‘induced fit’ mechanism of catalysis [26], [27]. By analysis of chimeric viral proteases of DEN2 and YFV, it was shown that the YFV polyprotein cleavage sites were efficiently cleaved by the chimeric protease containing the YFV or DEN2 NS3 protease domain, while the DEN2 polyprotein sites were not cleaved by the YFV chimeric protease containing YFV NS3(pro), suggesting that cleavage requires specific local interactions between substrates and the binding pocket site of the enzyme [28]. The substrate recognition sequence is highly conserved in all flaviviruses and consists of two basic residues in P2 and P1 followed by a small unbranched amino acid in P1’ [22], [43]. Substrate profiling studies found that the WNV protease was highly selective for the cleavage site sequence motif (K/R)↓GG, whereas DEN protease also tolerated the presence of bulky residues such as Phe, Trp, or Tyr at either the P1’ or the P2’ site, provided that the other position was occupied by Gly [21], [28], [29].

The aim of this study was to develop a fast and easy methodology for cloning, expression and purification of the active JEV NS2B(H)-NS3 serine protease and to obtain numerical data for kinetic constants by using ﬂuorogenic model peptide substrates for serine proteases. In addition, we also characterized inhibition of the protease by conventional serine protease inhibitors. To the best of our knowledge, this is the first study into the biochemical characteristics, substrate preferences and inhibitor profile of the protease encoded in the JEV genome.

## Materials and Methods

### Construction of JEV NS2B(H)-NS3pro Expression Plasmid

The full-length sequence of JEV genotype III strain JaOArS 982 NS2B-NS3 (Genebank accession number: M18370) was custom-synthesized (Blue Heron Technology Inc., Canada) and cloned into pLS vector (Top Gene Technologies, Canada). In close analogy with procedures previously published for DEN NS2B(H)-NS3pro [30], the pLS/NS2B-NS3 plasmid template was used to PCR-amplify the NS2B(H) region (amino acids 51–95 and 121–131 using specific primers NS2B(H)-F:

5′-GGATCCGTGTCAGGAAAAGCAACAGATATGTGGCTTGAACGGGC-3′,

(underlined sequence represents the *Bam*H1 restriction site) and

NS2B(H)-R 5′-**GCCCCCTCTTTTTGTTGTTTTTAAAGTGAGCCAATAACC**TGGAA CACCGGGAT CATCAATC-3′,

where the overlapping sequence is in bold; the NS3(pro) region (amino acids 1–180) using primers

NS3(pro)-F

5′-CCCGGTGTTCCA**GGTTATTGGCTCACTTTTAAAAACAACAAAAAGA-**GGGGGCGTGTTTTGGGACACGC-3′,

(overlapping sequence in bold letters);

and NS3(pro)-R

5′-GGTACC *CTA*TCTCTTTCTCAACATGTTTGGGGTGTAAGC-3′

(underlined sequence represents the *Kpn*1 restriction site and the sequence in italic indicates the introduced stop codon).

All oligonucleotides were purchased from Proligo Singapore Pte Ltd.

PCR was performed by using a thermal cycler GeneAmp PCR system Model 2400 (Perkin Elmer Cetus, USA), with pre-denaturing (95°C, 5 minutes), 25 cycles of denaturing (95°C, 60 seconds), annealing, (50°C, 30 seconds), extension (72°C, 2 minutes), and final extension (72°C, 8 minutes).

The NS2B(H)-NS3(pro) fragments containing NS2B residues 51–95 followed by residues 121–131, and the first 180 residues of NS3(pro) were constructed from the full-length sequence by overlapping extension PCR. PCR products harboring overlapping sequences, NS2B(H) and NS3(pro), were combined and subjected to splicing by overlap extension PCR (SOE-PCR) using primer pairs, NS2B(H)-F and NS3(pro)-R. The resulting SOE-PCR products were separated by agarose gel electrophoresis, and the 800 bp fragment was excised and purified by QIAquick® gel extraction kit (QIAGEN, Germany).

The purified PCR products and pTrcHisA (Invitrogen, USA) vector were digested with restriction enzymes *Bam*H1 and *Kpn*1 and purified by QIAquick purification kit. Insert DNA and pTrcHisA vector were combined at a 16∶1 molar ratio in a ligation reaction containing 1 × ligation buffer (50 mM Tris-HCl, pH 7.6, 10 mM MgCl_2_, 1 mM ATP, 1 mM DTT, 25% (w/v) polyethylene glycol 8000) and five units of T4 DNA ligase (Gibco BRL, USA) in a final volume of 20 µl and incubated overnight at 14°C, resulting in NS2B(H)-NS3pro carrying a N-terminal (His)_6_ purification tag.

The sequence of the recombinant (His)_6_-NS2B(H)-NS3pro construct was confirmed by DNA sequence analysis (Macrogen Inc., South Korea), using the sequencing primers, pTrcHis-F: 5′-GAGGTATATATTAATGTATCG-3′; and pTrcHis-R: 5′-CTGAAAATCTTCTCTCATC-3′.

### Expression and Purification of NS2B(H)-NS3pro

*Escherichia coli* DH5α (GIBCO BRL, USA) was used as host cell for plasmid propagation. *E. coli* C41 (F^−^, *omp*T, *hsd*SB, (r_B_^−^ m_B_^−^), *gal, dcm*, λ(DE3)), derived from *E. coli* C41 (DE3), was used as expression host. Constructs were transformed into *E. coli* (BL21) and cells were grown in one liter LB medium containing 100 µg ml^−1^ ampicillin, at 37°C, until OD_600_ reached 0.6. Expression was induced by isopropyl β-D-1-thiogalactopyranoside (IPTG) at a concentration of 0.2 mM and cells were incubated for 15 h at 18°C. Cells were harvested by centrifugation (6000 × g, 4°C, 10 minutes) and the pellet was resuspended in 30 ml lysis buffer (0.1 M Tris-HCl, pH 7.5, 0.3 M NaCl, 0.25 mg ml^−1^ lysozyme, 10 µg ml^−1^ DNase, and 5 mM MgCl_2_). Cells were kept at room temperature for 30 minutes and then lysed on ice by sonication using an Ultrasonic Processor XL (Misonix Inc. NY). Insoluble material was pelleted by centrifugation (15000 × g, 4°C, 20 minutes), and the soluble fraction was filtered through 0.22 micron filters (Pall Corporation, USA). Histidine-tagged NS2B(H)-NS3pro was purified by immobilized metal ion affinity chromatography (IMAC). Nickel-sepharose HisTrap™ HP 5 ml columns (GE Healthcare, Sweden) were pre-equilibrated with 10 column volumes sample buffer (0.1 M Tris-HCl, pH 7.5, 0.3 M NaCl) and the sample (30 ml from 1.0 liter of bacterial culture) was loaded at a flow rate of 1 ml min^−1^, using an FPLC pump (ÄKTA™FPLC™ system, GE Healthcare). The column was washed with 10 column volumes of degased washing buffer (0.1 M Tris-HCl, pH 7.5, 0.3 M NaCl, 30 mM imidazole). Protein was eluted with ten column volumes elution buffer (0.1 M Tris-HCl, pH 7.5, 0.3 M NaCl, 0.3 M imidazole) at flow rate of 1.0 ml min^−1^. Elution was monitored by absorbance at 280 nm using a UV detector (ÄKTA™FPLC™ system, GE Healthcare) and fractions of 1.0 ml were collected. Aliquots of 20 µl from each fraction were loaded onto a 15% SDS-PAGE gel and electrophoresis was performed in Tris-glycine buffer (25 mM Tris-HCl, pH 8.3, 192 mM glycine and 0.1% SDS). The gel was stained with Coomassie-Blue staining solution (0.1% Coomassie-Brilliant-Blue R250, 50% methanol and 10% glacial acetic acid) with shaking at room temperature for 2 hours, and then destained with shaking in destaining solution (10% methanol and 10% glacial acetic acid) at room temperature, overnight. Western blotting was performed using anti-hexahistidine antiserum (Invitrogen, CA, USA) at 1∶10,000 dilution. Fractions containing NS2B(H)-NS3pro were desalted by step-wise dialysis at 4°C by using SPECTRA/POR dialysis membranes (6–8 kDa MWCO) (Spectrum Medical Industries, Inc. MA, USA), against three batches of a 100-fold sample volume buffer A (0.1 M Tris-HCl, pH 8.5, 0.2 M NaCl), one batch of a 200-fold volume buffer B (0.1 M Tris-HCl, pH 8.5, 0.1 M NaCl, 5% (v/v) glycerol) and one batch of a 200-fold volume of buffer C (0.1 M Tris-HCl, pH 9.5, 5% (v/v) glycerol). Purified NS2B(H)-NS3pro was further concentrated to 1.0 mg ml^−1^ by centrifugal filter devices (Centricon 15 ml, 5 kDa MWCO, Millipore, USA) at 4°C. Protein concentrations were determined with a Bradford protein micro-assay using (Bio-Rad, USA) with bovine serum albumin (Sigma Chemistry) as calibration standard. Samples were stored in 50 mM Tris-HCl, pH 9.0, (50% v/v) glycerol, at −20°C.

### Assay of Enzymatic Activity

Enzymatic activity of purified NS2B(H)-NS3pro was assayed against commercially available synthetic peptide substrates containing either three non-prime side residues, Boc-GRR-amc and Boc-LRR-amc; four residues, Ac-nKRR-amc, Ac-nKRR-amc; and five residues, Pyr-RTKR-amc, and a previously described internally quenched DEN NS3 substrate, Abz-(R)_4_SAGnY-amide [22] (all purchased from Peptides International, KY, USA). Cleavage of amc from the peptide substrates was monitored on a microtiter plate fluorometer using a Beckman Coulter DTX 880 multimode reader (Beckman Coulter, CA, USA) at an excitation wavelength (λ) = 360 nm and an emission wavelength (λ) = 485 nm for all substrates, except for Abz-(R)_4_SAGnY-amide (excitation wavelength (λ) = 320 nm and emission wavelength (λ) = 420 nm). Assays were conducted on 96-well flat bottom black polystyrene microplates (Corning Life Sciences, MA, USA) in a reaction volume of 100 µl containing 0.5 µM NS2B(H)-NS3pro, assay buffer (50 mM Tris-HCl, pH 9.5, 30% glycerol) and substrate at concentrations ranging from 2.5 to 1500 µM. Reaction mixtures were pre-incubated for 30 min at 37°C and started by addition of the substrate. Fluorescence release was monitored every 30 seconds over a period of 5 min and relative fluorescence units were converted to rates of product formation by calibration with free amc (Sigma Chemistry, St. Louis, USA). Inner filter effects were corrected for as described in the literature [31]. Reaction velocities at steady state were calculated from the slope of reaction progression curves by non-linear regression of initial velocities using Graphpad Prism software. Kinetic parameters, *K*_m_, *k*_cat_ and catalytic efficiency *k*_cat_/*K*_m_, were calculated assuming Michaelis-Menten kinetics, v = V_max_(S)/(S)+*K*_m_). No significant hydrolysis of the peptide substrates was observed in the absence of enzyme.

The effect of pH on enzymatic activity on the JEV protease was determined by assays in different buffers in the pH range from 6.5–11.0 (50 mM MES, pH 6.5; 50 mM MOPS, pH 7.0; 50 mM Tris-HCl, pH 7.5, 8.0, 8.5, 9.0 and 9.5, and 50 mM CAPS, pH 10.0, 10.5 and 11.0) with 20% (v/v) glycerol in each assay using 100 µM Ac-nKRR-amc as substrate. The effect of ionic strength on enzyme activity was examined in assay reactions containing 0–200 mM NaCl in 50 mM Tris-HCl, pH 9.5, 30% (v/v) glycerol, 0.5 µM enzyme and 100 µM Pyr-RTKR-amc substrate.

Enzyme inhibition was characterized by using aprotinin (Sigma Chemistry, USA) as model inhibitor. JEV NS2B(H)-NS3pro (0.5 µM) was incubated in assay buffer (50 mM Tris-HCl pH 9.5, 20% (v/v) glycerol) in the presence of increasing concentrations of aprotinin (0–20 µM) at 37°C for 30 minutes. Reactions were started by adding 10 µM Pyr-RTKR-amc substrate. IC_50_ values for aprotinin were determined from dose-response curves. Experiments were performed in triplicate and the standard deviation of all reported numerical values was <10% with exception of the Boc-LRR-amc peptide substrate where SD was 12.5%.

### Sequences Alignment and Homology Modelling

Amino acid sequences of the NS2B-NS3 of WNV, JEV, DEN2 and YFV (UniProt IDs: P06935, P27395, P2999 and Q6DV88) were imported from the Universal Protein Resource, UniProt [32]. Multiple sequence alignment of the four NS2B-NS3 proteases was done with ClustalW, realized in UniProt.

The crystal structures of the WNV protease/inhibitor complex (NS2B(H)-NS3-protease-Bz-nKRR-H, PDB ID: 2FP7) was used as template to build a model of the JEV NS2B(H)-NS3-protease. A pairwise sequence alignment was first done for the WNV and JEV NS2B-NS3 proteases using the ClustalW algorithm of UniProt. Homology models were then built using Modeller 9.9 [33]. The model with the lowest DOPE Score was chosen as the best.

## Results

### 3.1. Cloning, Expression and Purification of JEV NS2B(H)-NS3pro

**Figure 1:**
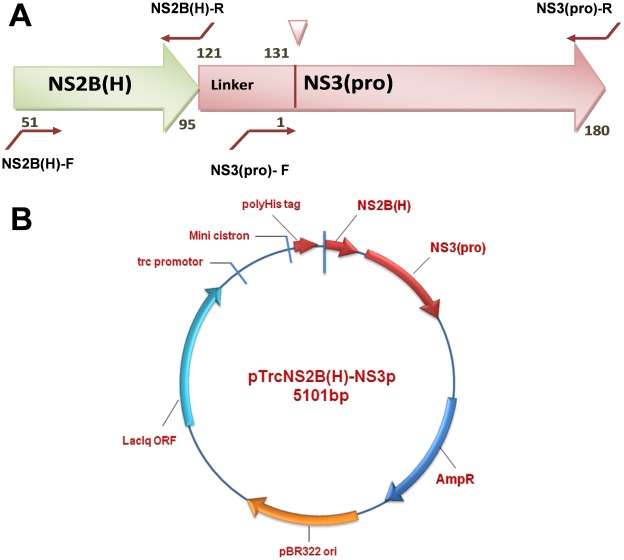
Schematic representation of primer binding sites and physical map of pTrcHisA/NS2B(H)-NS3(pro). (A) The figure illustrates the NS2B(H)-NS3(pro) fragment and the primer binding positions. The NS2B(H), NS2B C terminal 11 amino acid residues linker and NS3 protease domains are shown. The NS2B-NS3 cleavage site is represented as a triangle. PCR primers are shown in maroon lines representing overlapping sequences. Amino acid positions within NS2B and NS3 are shown as black letters. (B) Shown is the recombinant plasmid, pTrcHisA/NS2B(H)-NS3(pro) of JEV encoding the 31 kDa NS2B(H)-NS3(pro) from JEV. The plasmid backbone contains the *trc* promoter (pTrc), *lac* operator (*lac*O), polyhistidine (His)_6_ tag, Xpress™ epitope (Xpress), ampicillin resistance gene (AmpR) and lacI^q^ repressor genes (lacIq). The plasmid map was generated by the Vector NTI program.

**Figure 2:**
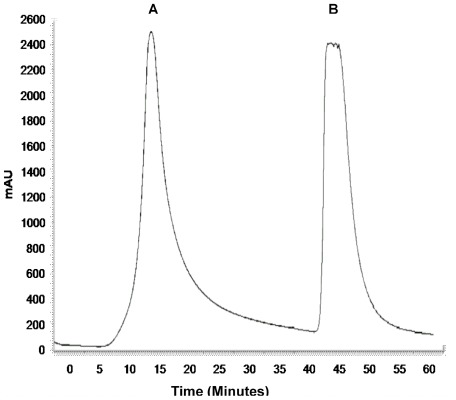
Purification profile of JEV NS2B(H)-NS3(pro) by Ni^2+^ affinity column. This figure shows a chromatogram from the purification of JEV NS2B(H)-NS3(pro) by Ni^2+^ affinity chromatography The column was washed with 0.1 M Tris-HCl, pH 7.5, 0.3 M NaCl, 30 mM imidazole (peak A) and NS2B(H)-NS3pro was eluted with elution buffer (0.1 M Tris-HCl, pH 7.5, 0.3 M NaCl, 0.3 M imidazole (peak B).

**Figure 3:**
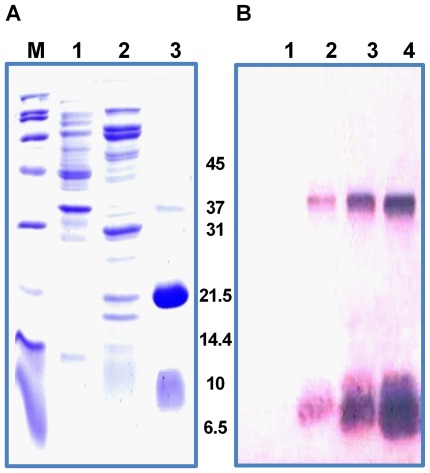
SDS-PAGE and Western blot analysis of NS2B(H)-NS3(pro). (**A**) Samples from the IMAC column were loaded onto a 15% SDS-PAGE gel and electrophoresis was performed in Tris-glycine buffer. The gel was stained with Coomassie-Blue. Lane M, broad range protein marker; lane 2, IMAC flow-through; lane 3, 30 mM imidazole wash peak; lane M imidazole elution peak. (**B**) Western blot profile using N-terminal anti-His antibody on 15% SDS-PAGE. Lane 1, 30 mM imidazole peak fraction; lane 2, 0.3 M imidazole peak fraction; lane 3, 0.3 M imidazole after dialysis (desalting); lane 4, 0.3 M imidazole peak fraction after dialysis and concentrating (Centricon, Millipore). The band representing the intact protein shows the highest intensity upon induction of expression.

Starting from an *in vitro* synthesized gene sequence encoding the full-length NS2B-NS3 protein from JEV, an enzymatically active single-chain protease NS2B(H)-NS3pro was constructed by SOE-PCR and cloned downstream of an N-terminal hexahistidine purification tag into expression vector pTrcHisA (Fig. 1). Recombinant plasmid DNA was transformed into *E. coli* DH5α followed by rapid size screening and restriction digestion analysis. The complete sequence of the cloned JEV NS2B(H)-NS3pro was analyzed by automated DNA sequencing in both forward and reverse directions, and resulting sequences were compared to the nucleotide sequence of JEV genotype III strain JaOArS 982 [Genebank accession number M18370.1]. No premature stop codons or amino acid substitutions were introduced in the recombinant sequence. NS2B(H)-NS3pro was expressed upon incubation for 12 h in the presence of 0.2 mM IPTG, predominantly as a soluble protein and was purified to >95% purity by a single-step chromatography on Ni^2+^ - metal chelate affinity columns, eluting at 0.3 M imidazole (Fig. 2). SDS-PAGE analysis and subsequent Western immunoblotting with anti-polyhistidine antibodies of the purified protein revealed the presence of two major proteins with molecular weights 21 and 10 kDa, and a relatively faint band at 36 kDa, thereby suggesting almost complete autocleavage of the enzymatically active NS2B(H)-NS3pro protease at the native NS2B/NS3 cleavage site (Fig. 3).

### 3.2 Enzyme Assay with Fluorogenic Peptide Substrates

**Figure 4:**
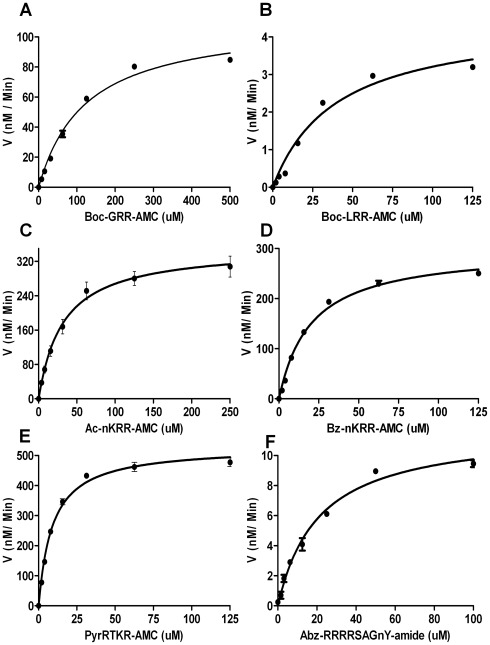
JEV NS2B(H)-NS3(pro) catalysed substrate hydrolysis rates at different substrate concentrations. The graph shows reaction velocities of JEV NS2B(H)-NS3(pro) for hydrolysis of Boc-GRR-amc (A); Boc-LRR-amc (B); Ac-nKRR-amc (C), Bz-nKRR-amc (D), Pyr-RTKR-amc (E), and Abz-(R)_4_SAGnY-amide (F). Assays were performed at 37°C in 50 mM Tris-HCl, pH 9.5, 30% v/v glycerol. Kinetic parameters were determined by non-linear fitting of untransformed data to the Michaelis-Menten equation. Data are reported as the mean of three experiments ± standard error (SEM).

Enzymatic activity of the recombinant NS2B(H)-NS3pro protein was assayed by fluorescence release from several small synthetic peptide substrates resembling the dibasic cleavage site sequences of the JEV polyprotein precursor. In addition, we have analysed the activity of JEV NS2B(H)-NS3pro by using a tetrabasic internally quenched fluorescent peptide, Abz-(R)_4_SAGnY-amide, originally described for the DEN NS3 serine protease [22]. Peptide substrates labelled with the amc reporter group comprised three to five amino acid residues at the non-prime side, whereas the tetrabasic substrate contained three residues added to the prime side of the peptide cleavage sequence. Initial velocities for each substrate (RFU min^−1^) were converted to concentrations of released amc and velocities (nm min^−1^) were re-plotted as a function of substrate concentration (Fig. 4). Upon correction of inner filter effects, data were in excellent agreement with Michaelis-Menten kinetics, as revealed by non-linear least square fit of data, and kinetic parameters, *K*_m_, *k*_cat_, and catalytic efficiency *k*_cat_/*K*_m_, were determined for each substrate (Table 1).

**Table 1:** JEV NS2B(H)-NS3 Kinetic Parameters for Fluorogenic Substrates.

Substrate	*K*_m_(µM)	*k*_cat_(s^−1^)	*k*_cat_/*K*_m_(M^−1^s^−1^)
Boc-GRR-amc	123±11.6	0.0037±0.0002	30.08±2.7
Boc-LRR-amc	40±5.0	0.00015±0.0001	3.74±0.3
Ac-nKRR-amc	32±2.5	0.01178±0.00054	368.13±17.0
Bz-nKRR-amc	20±1.34	0.01±0.00023	500.00±11.3
Pyr-RTKR-amc	9.0±0.82	0.0176±0.0008	1962.96±85.0
Abz-(R)_4_SAGnY-amide	20.91±2.04	0.00040±0.000014	19.000±1.85

Sequences of the peptides analysed showed differing substrate binding activities (*K*_m)_ as well as substrate turnover (*k*_cat_) and kinetic data obtained for the substrates displayed relatively large variations ranging from *k*_cat_/*K*_m_ 3.74 M^−1^ s^−1^ (Boc-LRR-amc) to 1,963 M^−1^ s^−1^ (Pyr-RTKR-amc). The most inefficient substrate (based on *k*_cat_/*K*_m_) was Boc-LRR-amc, with a low *k*_cat_ of 0,00015 s^−1^. Although the second least efficiently cleaved amc-labeled substrate, Boc-GRR-amc, showed a 3-fold higher *K*_m_ (123 µM), turnover was approximately 10-fold greater than for Boc-LRR-amc. In earlier studies Boc-GRR-amc has shown *K*_m_ of 142 µM and *k*_cat_ of 0.034 s^−1^ on NS2B/NS3 protease of YFV and *K*_m_ of 150 µM and *k*_cat_ of 0.13 s^−1^ on NS2B/NS3 protease of DEN2 [34], [35] Thus, this substrate containing P2-P1 Arg-Arg as in cleavage sites of YFV and DEN2 viruses seem to be bound equally tight, but cleaved with lower turnover by JEV protease than by YFV and DEN2 proteases.

The most efficiently cleaved substrate, Pyr-RTKR-amc, had the lowest *K*_m_ (9 µM) and the highest turnover number (0.0176 s^−1^) of all amc-labelled peptides. This substrate has been described in an earlier study on DEN2 NS2B-NS3 protease where values of 134 µM, 0.013 s^−1^ and 97 M^−1^ s^−1^ were reported for *K*_m_, *k*_cat_ and *k*_cat_/*K*_m_ respectively [24]. It was also described in another study on N2B-NS3 proteases of WNV and DEN2, where the *K*_m_, *k*_cat,_ and *k*_cat_/*K*_m_ values were 71 µM, 6.3 s^−1^ and 88000 M^−1^ s^−1^ for WNV protease and 3.6 µM, 0.02 s^−1^ and 5500 M^−1^ s^−1^ for DEN2 [29]. Data obtained for *k*_cat_/*K*_m_ with the substrate peptides Ac-nKRR-amc and Bz-nKRR-amc revealed a discernible contribution of the N-terminal protection group on the activity of the respective substrate, whereby the benzoyl moiety contributes to an approximately 1.4-fold higher *k*_cat_/*K*_m_ when compared to the acetyl group.

Although the internally quenched substrate Abz-(R)_4_SAGnY-amide was shown previously to be efficiently cleaved by DEN NS2B(H)-NS3pro, (*k*_cat_ */K*_m_: 11087 M^−1^ s^−1^) [22], binding affinity and cleavage efficiency (*k*_cat_ */K*_m_: 19 M^−1^ s^−1^) of this peptide was substantially lower (approx. 580-fold) for JEV NS2B(H)-NS3 protease than for the DEN NS3 protease. This finding is in agreement with notable differences in substrate preferences between JEV and DEN NS3 as assumed from a comparison of cleavage site sequences present in the viral polyprotein precursor [29].

**Figure 5:**
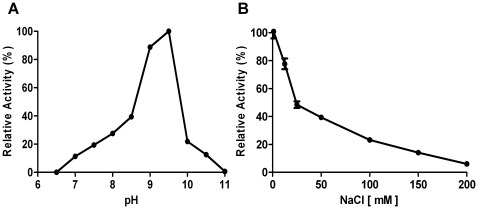
Effect of pH and ionic strength on the proteolytic activity of NS2B(H)-NS3(pro) from JEV. (**A**) Shown are activities as assayed by using 150 µM Ac-nKRR-amc substrate at 37°C at pH ranging from 6.5–11.0 by using different buffers (50 mM MES, MOPS, Tris, CAPS). (**B**) Ionic strength dependence of JEV NS2B(H)–NS3(pro) enzyme activity. Assay was performed at 37°C using 100 µM Pyr-RTKR-amc, 0.5 µM enzyme and 50 mM Tris-HCl pH 9.5 buffer containing 20% glycerol (v/v) in the presence of increasing salt concentrations (0–200 mM).

The effect of pH on the enzymatic activity of JEV protease was determined by assays using buffers in the pH range from 6.5–11.0 (Fig. 5, panel A). In agreement with earlier reports on flaviviral proteases [15], [24], [25], [36] the pH optimum for reaction with the peptide substrate Ac-nKRR-amc was 9.5. It is noteworthy that the activity of the enzyme at physiological pH is less than 25% of the activity observed at pH 9.5.

Flaviviral serine proteases exhibit marked inhibition by high salt concentrations [24], [25], and we examined effects of elevated ionic strength by high concentrations of NaCl (Fig. 5, panel B). Cleavage of the substrate Pyr-RTKR-amc was inhibited by about 50% in the presence of 25 mM NaCl, which can be compared to the 40–50% inhibition of the dengue virus NS3 protease caused by 100 mM NaCl, as earlier reported [24], [37], thereby suggesting an even greater sensitivity of the JEV NS3 and MVEV proteases to high ionic strength.

**Figure 6:**
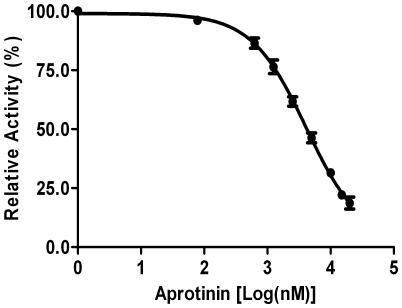
JEV NS2B(H)-NS3 Inhibition Dose-Response Curve. Inhibition of NS2B(H)-NS3 protease activity mediated by aprotinin. The inhibition was performed by incubation of 0.5 µM of NS2B(H)-NS3 in assay buffer (50 mM Tris-HCl, pH 9.5, 20% glycerol) with increasing concentrations of aprotinin (0.0–20 µM). The results are representative of three independent experiments.

Earlier studies have shown that the activities of flaviriral proteases from DEN and WNV are comparatively insensitive to inhibition by conventional protease inhibitors, like PMSF and benzamidine [25], [47]. We here evaluated the inhibitory activities of aprotinin on the JEV protease. Dose-response plots in the presence of increasing inhibitor concentrations assayed with the Pyr-RTKR-amc substrate suggest IC_50_ values of 4.13±0.17 µM for aprotinin (Fig. 6). Previous studies have reported *K*_i_ values for aprotinin of 0.162 µM for WNV NS3 protease, and 0.026 µM for DEN2 NS3 protease [15]. Similarly, for MVEV NS2B-NS3, the IC_50_ of aprotinin is about 8 µM [14]. In another study, using a non-cleavable form of WNV NS2B-NS3pro, the *K*_i_ of aprotinin was reported as 26 nM [38].

Thus, in comparison, JEV NS3 protease appears to be even less susceptible to this protease inhibitor evaluated here.

### 3.3 Structural Comparisons of JEV Protease with WNV, DEN and YVF Proteases

**Figure 7:**
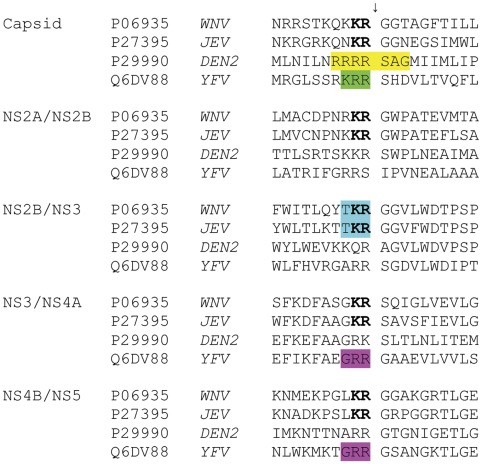
Multiple sequence alignment of WNV, JEV, DEN and YFV cleavage junctions recognized by the NS2B-NS3 protease. The arrow above the sequences shows the scissile bond cleaved by the protease. The sequences which served as basis for the fluorogenic substrates used herein are marked with colors (Abz-(R)_4_SAGnY-amide (yellow), Ac-nKRR-amc and Bz-nKRR-amc (green), Pyr-RTKR-amc (blue), and Boc-GRR-amc (purple). The conserved amino acids Lys-Arg at the P2-P1 positions in JEV and WNV are marked in bold font.

The alignment of the JEV, WNV DEN2 and YFV polyprotein sites cleaved by NS2-NS3 proteases is shown in Fig. 7. As seen, the JEV and WNV sites are the most similar; they all contain Lys-Arg at the P2-P1 positions, while at the P’1 position a Gly is present at four out of the five sites (the exception is the NS3/NS4A-junction, which contains a Ser). The DEN2 and YFV sites are less similar with those of JEV, however; in six cases out of ten, these sites have Arg-Arg at the P2-P1 positions, while the four remaining sites have different positively charged residues (Lys-Arg, Arg-Ser, Gln-Arg and Arg-Lys) at these positions. It is also notable that the five cleavage sites of JEV contain five different amino acids at P3; the same holds true for P4.

**Figure 8:**
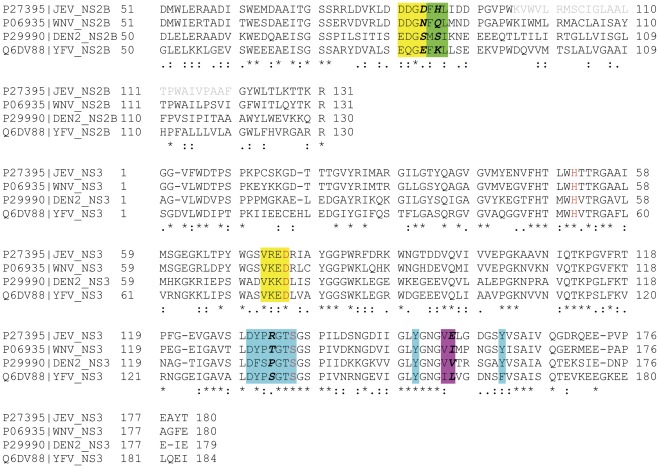
Multiple sequence alignment of the NS2B-NS3 protease of WNV, JEV, DEN and YFV. Residues located in the S1, S2, S3 and S4 pockets are marked in colours cyan, yellow, green and magenta, respectively (data from [39]). Non-conserved residues located at the binding pockets are marked by bold italic font.

The multiple sequence alignment of the WNV, JEV, DEN2 and YFV NS2B-NS3 proteases is shown in Fig. 8. The alignment revealed that the JEV protease is closest to WNV, with a sequence identity of 76.3% (93.7% sequence similarity), while the DEN2 and YFV proteases show only 50.2% and 45.5% sequence identity (79.5% and 76.9% sequence similarity) with the JEV protease, respectively.

The X-ray crystal structure of the WNV protease/inhibitor complex NS2B(H)-NS3-protease-Bz-nKRR-H [27] indicates that the WNV NS2B-NS3 protease has four distinct substrate binding pockets, termed S4-S1, which accommodate the P4-P1 residues of protease substrates [39]. The S1 pocket is composed of nine residues; the key interactions with P1 Arg are being formed by the side chain of Asp129 and the backbone carbonyl oxygen of the Tyr130 residue of NS3. A large part of the S1 pocket comprises the aromatic side chains of Tyr150 and Tyr161 of NS3. In the X-ray structure Tyr161 is forming a cation-π stacking with P1 arginine, which is located between Tyr161 and Bz cap of the inhibitor; possible cation-π stacking of substrate P1 arginine with Tyr150 is also suggested from mutagenesis studies [40], [48]. The S2 pocket of the WNV protease is outlined by eight residues and is dominated by a negative electrostatic potential from backbone carbonyl oxygen atoms of Asp82 and Gly83 of NS2B, and Val71 and Lys72 of NS3, as well as from acidic side chains of Asp82 of NS2B and Asp75 of the catalytic triad. S3 and S4 pockets are less well defined and consist of a few (three and two, respectively) uncharged amino acids, which makes the substrate P4 and P3 residues being largely solvent exposed. The sequence stretches contributing to the S4-S1 pockets are marked in the multiple alignment of Fig. 8. As seen, JEV and WNV share the same amino acid at 17 of the 22 indicated positions; JEV and DEN2 share the same amino acid at 11 positions and JEV, and YFV at 12 positions. However, there are positions in each one of S4-S1 pockets, which contain physico-chemically quite different amino acids. Residue 84 of NS2B contributes to the S2 pocket, and is Asp, Asn, Ser and Glu in, respectively, JEV, WNV, DEN2 and YFV. Residue 86 of NS2B is part of the S3 pocket and contains also four different amino acids in the four viruses (His, Gln, Ser, and Lys). Positions 132 and 155 of NS3, which belong to S1 and S4 pockets, respectively, are also different in the different proteases. These amino acid differences are thus likely candidates to contributing to the differences in kinetics of substrates for the different proteases.

**Figure 9:**
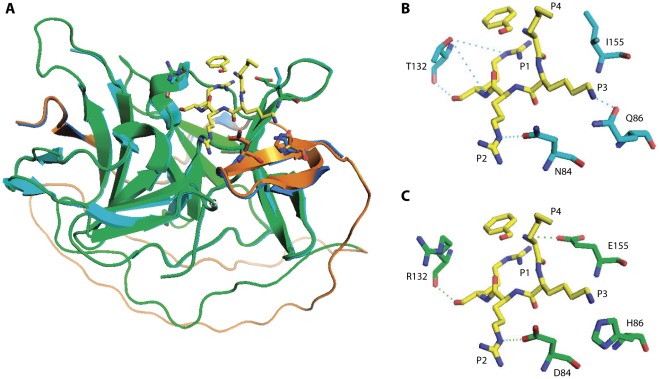
Structures of JEV and WNV proteases bound to Bz-nKRR-H. (**A**) Superimposition of the WNV X-ray structure (PDB ID 2FP7) on the JEV homology model. The WNV structure is shown in blue (NS2B) and cyan (NS3); the JEV structure in orange (NS2B) and green (NS3). The Bz-nKRR-H inhibitor in the WNV structure is shown in stick representation in yellow. (**B**) Detailed interactions of Bz-nKRR-H with WNV (from X-ray structure). (**C**) Superimposition of the Bz-nKRR-H X-ray structure (PDB ID 2FP7) on the JEV homology model. Potential hydrogen bonds indicated by dashed lines. Putative interacting amino acid residues of WNV and JEV are represented in cyan and green, respectively, and Bz-nKRR-H in yellow.

In order to cast further light into this possibility, we built a homology model of the JEV NS2B-NS3 protease, using the crystal structure of a WNV NS2B(H)-NS3-protease-Bz-nKRR-H inhibitor complex as template. The model for the JEV protease is shown superimposed on the WNV protease/inhibitor complex in Fig. 9, panel A. Pairwise 3D structure alignment showed that the JEV and WNV proteases share large structural similarity (RMSD = 0.172 Å), with a large structural conservation in the enzymes’ active sites. However, clear differences are seen in the substrate-binding pockets at NS2B residues Asn84, Gln86, and NS3 residues Thr132, Ile155 (WNV NS2B-NS3 protease numbering, corresponding to NS2B residues Asp84, His86, and NS3 residues Arg132, Glu155 in the JEV protease). This is visualized in Fig. 9, panels B and C. Side chains of all four residues are in close proximity with the ligand and are capable of forming multiple hydrogen bonds (except Ile 155 in the WNV protease). The superimposition shows that the change of these residues between JEV and WNV leads to a serious rearrangement of the H-bond network in the substrate-binding pocket, which very likely will affect the cleavage preferences of the two proteases. Moreover, while all these residues are uncharged in the S1-S4 pockets of the WNV protease, the corresponding residues in the S2 and S4 pockets of the JEV protease are acidic (Asp and Glu) while in the S1 pocket it is basic (Arg). All of this may influence both the ionic interactions with substrates and the geometries of the binding pockets.

## Discussion

Over the past decade, the NS2B-NS3 two-component serine proteases of human-pathogenic flaviviruses have received substantial scientific interest as potential drug targets, as these enzymes are indispensable components of virus replication [13], [19], [41]. Compared to the NS3 proteases of the closely related DEN and WNV, the NS3 protein from JEV is much less well characterized. In this study we report a straightforward procedure for producing recombinant JEV NS2B(H)-NS3pro protease by overexpression in *E. coli* followed by an one-step purification procedure, and we report the kinetic parameters of the protease for commercially available synthetic fluorogenic model peptide substrates and serine protease inhibitors.

The full-length NS2B-NS3 polyprotein region of JEV was obtained by time- and cost-efficient gene synthesis, and was used as template for the generation of the NS2B(H)-NS3pro protease complex comprising residues 51–95 and residues 121–131 from the NS2B cofactor and N-terminal residues 1–180 of the NS3 protease domain. Previous studies have demonstrated that such a construct is catalytically active both in proteolytic autocleavage conducive to the formation of a non-covalent NS2B(H)-NS3pro complex and with peptide substrates supplied for *in trans* cleavage reactions [24].

The recombinant NS2B(H)-NS3pro protein produced by *E. coli* was predominantly biosynthesized as a soluble protein, and could be easily purified by metal chelate affinity chromatography to >95% purity. In SDS-PAGE analysis, the purified protein displayed bands of 36, 21 and 10 kDa, corresponding to the NS2B(H)-NS3pro precursor, the NS3pro protease domain and the histidine-tagged NS2B(H) cofactor, respectively. Similar to the protein from DEN, the 29.8 kDa (His)_6_NS2B(H)-NS3pro protein of JEV exhibits anomalous migration in SDS-PAGE gels. In contrast to the corresponding protein from DEN serotype 2, the NS2B(H)-NS3pro protein from JEV was largely soluble upon expression in *E. coli*, thus suggesting the existence of major conformational differences between the two proteins.

In analogy to a number of specificity studies on the NS3 proteases from DEN and WNV [15], [29], [42], [43], we assayed a small set of commercially available fluorogenic peptide substrates, based on P3-P1 sequences of cleavage sites (capsid protein, NS2B/NS3 itself, NS3/NS4A, and NS4B/NS5 cleavage site) of NS2B/NS3 proteases of WNV, YFV, and JEV. In addition, we assayed an internally quenched peptide, Abz-(R)4SAGnY-amide, representing the P4-P3' sequence of the capsid protein cleavage site of the DEN-2 virus [22].

Among all substrates used, the highest binding affinity and cleavage efficiency was observed for the Pyr-RTKR-amc substrate, which represents the native JEV NS2B/NS3 cleavage site at the P3 to P1 positions. The binding affinity for the substrate with a leucine at P3 (Boc-LRR-amc) was three-fold higher when compared to that with a glycine at P3 (Boc-GRR-amc); however, the catalytic efficiency for Boc-GRR-amc was about 10-fold higher than for Boc-LRR-amc, thereby suggesting a significant contribution of this position to the catalytic mechanism as seen in an earlier report for the DEN NS3 protease [44]. The LRR-amc substrate also displays the lowest catalytic efficiency of all tested peptides. Boc-GRR-amc and Boc-LRR-amc were previously assayed with the NS3 proteases from DEN and WNV, where it was found that both enzymes prefer a Gly residue to a Leu at the P3 position [15], [44]. Our results indicate that hydrolysis of substrates with a short chain amino acid (Gly) at the P3 position is favoured by the JEV NS2B(H)-NS3 protease over those with a bulky residue (Leu), which aligns with previous data reported for the DEN2 and WNV proteases [15], [45]. The prominent contribution to catalytic efficiency of residues at the P3 and P4 position (and possibly prime-side residues) for the JEV protease is also reflected by the comparatively weak activity of the internally quenched peptide Abz-(R)_4_SAGnY-amide, which is an efficient NS3 substrate originally designed from the capsid protein sequence RRRR▾SAG of DEN2 [22]; here it demonstrated a 580-fold lower *k*_cat_ */K*_m_ when assayed with the JEV enzyme, compared with DEN2. Whereas the tetrabasic non-prime side sequence is strongly favoured by DEN NS3, the presence of Asn and Gln at P3 and P4 of JEV apparently confers a high difference in specificity between the two enzymes. Moreover, it can not be ruled out that the prime-side sequences (SAG in DEN and GGN in JEV) contribute substantially to the decrease in efficiency as seen for this substrate peptide.

Although kinetic data for the JEV protease are relatively limited to date, it can be concluded that the enzyme from JEV favours substrates with Lys-Arg at the P2-P1 position, while the NS3 proteases from DEN and YFV prefer Arg-Arg at this position; data which thus suggest a greater similarity of the JEV protease to WNV than to DEN [15]. This view is supported by amino acid sequence alignments, the crystal structures for the enzymes from DEN and WNV, and structure-guided mutagenesis studies, which show that functional determinants of activation and substrate recognition for JEV and WNV are more closely related than other flaviviral proteases (Fig. 7). It is noteworthy that both the whole sequence and the substrate binding regions of the NS2B-NS3 protease JEV show much higher identity to WNV than to DEN2 and YFV proteases (Fig. 8 and [46]).

We carried out multiple sequence alignment of the NS2B-NS3 proteases of WNV, JEV, DEN and YFV, which allowed identifying differences in the putative binding pockets of the proteases (Fig. 8). We identified four amino acid residues (positions 84 and 86 in NS2B and 132 and 155 in NS3), which were different for all four proteases. In order to confirm the role of these residues in substrates binding and cleavage preferences of NS2B-NS3 proteases, we built a 3D homology model of the JEV NS2B-NS3 protease and compared its substrate binding pocket with that of the WNV NS2B-NS3 protease (Fig. 9). The structural modeling revealed that the conformation of the JEV protease is overall highly similar with the structure of the WNV NS2B-NS3 complex [21], [27], [45]. However, it can be seen that the change of Asn84, Gln86, Thr132 and Ile155 in WNV NS2B-NS3 protease to Asp84, His86, Arg132 and Glu155 in the JEV NS2B-NS3 protease leads to rearrangement of the possible H-bonds between the ligand and the S1, S3, and S4 pockets of the enzyme (Fig. 9, panel B, C). Moreover, presence of an acidic amino acid in S4 and a basic amino acid in S1 may influence both the binding pocket geometry and ligand-protease interactions. This makes the proteases of the two viruses quite different in terms of their binding mode, affinity and cleavage preferences and are likely reasons for the observed differences in the cleavage efficiency of substrates for the JEV and WNV enzymes.

An earlier study employing hybrid NS2B-NS3 constructs of DEN and JEV sequences showed that only a (DEN)NS2B-(JEV)NS3 protein could efficiently process the JEV polyprotein, whereas the (JEV)NS2B-(DEN(NS3) construct was inactive, supporting the notion that NS2B proteins of different origins modulate the structure and substrate affinity of the protease [21], [28]. This also indicates that there are likely differences in the conformational space between the NS2B-NS3 proteases of different flaviviruses.

The protein inhibitor aprotinin was found to inhibit JEV NS2B/NS3 protease with surprisingly low potency (IC_50_: 4.13±0.167 µM); this inhibitory efficiency is comparable with the previously reported inhibitory efficiency of aprotinin for the MVEV NS3 protease (IC_50_: 7.8±2.9 µM) [14]. It is noteworthy that there are several reports where differences in the susceptibility of the WNV protease to aprotinin was seen [48], [49], possibly suggesting a modulation of inhibitor sensitivity by recombinant sequences introduced by genetic engineering.

To sum up, in this study we present for the first time a comparative enzyme-kinetic analysis of a recombinant JEV protease by employing model substrate peptides. Our data demonstrate that the JEV protease shows marked differences to other known flaviviral proteases. Our study may serve as an entry point to the development of efficient JEV inhibitors, *e.g.* by employment of high-throughput screening.
